# Alcohol Consumption and Depressive Symptoms in Romanian University Students: Post-Pandemic Insights from a Non-Clinical Cohort

**DOI:** 10.3390/jcm15031314

**Published:** 2026-02-06

**Authors:** Daniela Gabriela Glavan, Madalina Aldea, Iulia Băluțoiu, Ramona-Constantina Vasile, Alexandra Daniela Rotaru-Zavaleanu, Sofia-Danai Dampa, Mihai Andrei Ruscu, Andrei Greșiță, Citto Iulian Taisescu, Eleftheria Dampa, Venera Cristina Dinescu

**Affiliations:** 1 Department of Psychiatry, University of Medicine and Pharmacy of Craiova, 2 Petru Rares Str., 200349 Craiova, Romania; daniela.glavan@umfcv.ro (D.G.G.); madalina.aldea@umfcv.ro (M.A.); 2 Department of Psychology, University of Medicine and Pharmacy of Craiova, 2 Petru Rares Str., 200349 Craiova, Romania; 3 Department of Epidemiology, University of Medicine and Pharmacy of Craiova, 2 Petru Rares Str., 200349 Craiova, Romania; mihai.ruscu@umfcv.ro; 4 Independent Researcher, 200349 Craiova, Romania; dansofd@gmail.com; 5 Department of Physiology, University of Medicine and Pharmacy of Craiova, 2 Petru Rares Str., 200349 Craiova, Romania; andrei.gresita@umfcv.ro (A.G.); citto.taisescu@umfcv.ro (C.I.T.); 6 Department of Internal Medicine, Kilkis General Hospital, Al. Gialamidi 2, 611 00 Kilkis, Greece; edampa@auth.gr; 7 Medical Research, Aristotle University of Thessaloniki, 541 24 Thessaloniki, Greece; 8 Department of Health Promotion and Occupational Medicine, University of Medicine and Pharmacy of Craiova, 2 Petru Rares Str., 200349 Craiova, Romania; venera.dinescu@umfcv.ro

**Keywords:** alcohol consumption, depression, university students, AUDIT, DASS-21, mental health, hazardous drinking

## Abstract

**Background:** University students are increasingly vulnerable to both depressive symptoms and hazardous alcohol use, particularly in the aftermath of the COVID-19 pandemic. Disruptions in circadian rhythms, hormonal dysregulation, and changing social dynamics may heighten susceptibility to maladaptive coping behaviors such as alcohol consumption. While this relationship has been widely studied in Western populations, limited data exist for Eastern European contexts. This study investigated the association between alcohol consumption and depressive symptoms among Romanian university students and explored potential gender differences in this post-pandemic cohort. **Methods:** A cross-sectional study was conducted among 103 Romanian university students at the University of Medicine and Pharmacy of Craiova, Romania. Participants anonymously completed a combined survey integrating the Alcohol Use Disorders Identification Test (AUDIT) and the Depression subscale of the Depression, Anxiety and Stress Scale (DASS-21). Statistical analyses included Pearson correlation, linear regression, and subgroup comparisons to evaluate associations between alcohol use and depression severity. **Results:** The mean AUDIT score was 5.4 ± 5.8, while the mean DASS-21 Depression score was 13.8 ± 9.5. A strong positive correlation was observed between AUDIT and depression scores (*r* = 0.72, 95% CI [0.62, 0.80], *p* < 1 × 10^−17^). Linear regression revealed that AUDIT scores significantly predicted depression severity (*R*^2^ = 0.496, *p* < 0.001), with each one-point increase in AUDIT score associated with a 1.31-point rise in depression score. Male students reported significantly higher alcohol use than females (*p* = 0.005), while depression scores did not differ significantly by gender (*p* = 0.110). The alcohol–depression association was similarly strong across genders. **Conclusions:** Hazardous alcohol use was highly prevalent and strongly associated with increased depressive symptoms among university students. These findings highlight the need for integrated mental health and substance use screening programs in university settings to support early identification and intervention.

## 1. Introduction

Depression and hazardous alcohol use are among the most pressing and burdensome public health concerns affecting university students worldwide [1]. The World Health Organization (WHO) estimates that more than 264 million people globally experience depression, with young adults representing a particularly vulnerable demographic as they navigate the psychosocial and academic transitions of higher education [2]. This life stage often coincides with increased autonomy, exposure to peer influences, and heightened academic pressures, factors that may contribute to maladaptive coping strategies, including problematic alcohol use [3]. Across Europe, North America, and Asia, studies consistently show high rates of binge drinking and risky alcohol consumption among university populations, often exceeding those observed in older adult groups [4].

Cross-cultural studies have demonstrated considerable variability in the prevalence and correlates of alcohol use and depression among university students. Research conducted in Ethiopian university students reported high rates of both depression and substance use, with significant associations between these conditions [5,6]. Similarly, studies among German university students have examined social norms and interventions targeting heavy drinking, highlighting the widespread nature of hazardous alcohol consumption in European academic settings [7]. In Asian contexts, research has documented distinct patterns of drinking behavior influenced by cultural norms and academic pressures [8]. Studies from the United States have consistently shown high comorbidity between depressive symptoms and alcohol misuse in college populations, with gender-specific patterns in help-seeking behavior [9]. Despite this growing body of international literature, Central and Eastern European student populations remain underrepresented, with limited data available from countries such as Romania where cultural attitudes toward alcohol and mental health stigma may differ substantially from Western contexts [10,11]. These differences have been further accentuated by the COVID-19 pandemic, which constituted a major disruption in the developmental trajectory of today’s young adults [11]. In Romania, the pandemic unfolded against a distinctive socio-political and economic backdrop, compounding vulnerabilities and shaping behavioral outcomes such as alcohol consumption [12]. Although the present study does not analyze pandemic influence directly, this context remains a crucial backdrop for understanding emerging health trends in this region. To better understand the mechanisms underlying these observed behavioral patterns, it is important to explore the bidirectional relationship between depression and alcohol misuse.

Emerging evidence also suggests that pandemic-related lifestyle disruptions, such as altered sleep–wake cycles, increased screen time, and reduced social and daylight exposure, have led to dysregulation in circadian rhythms and hormonal functioning [13]. Specifically, changes in melatonin secretion and elevated cortisol levels have been associated with emotional dysregulation, impaired cognitive functioning, and greater susceptibility to maladaptive coping behaviors such as alcohol use [14]. These physiological disruptions may underlie both depressive symptomatology and increased alcohol consumption in the post-pandemic period, offering a complementary explanatory framework for the behavioral patterns observed among university students.

Both depression and excessive alcohol use exert substantial individual and societal costs. Depression impairs cognitive functioning, motivation, and social relationships, while alcohol misuse increases the likelihood of accidents, violence, and poor academic outcomes. Crucially, these two conditions frequently co-occur [15]. The comorbidity between depressive symptoms and alcohol misuse has been attributed to shared etiological pathways, such as dysregulation in serotonergic and dopaminergic systems, genetic predisposition, and overlapping environmental stressors [16]. The self-medication hypothesis posits that individuals experiencing depressive affect may use alcohol to alleviate negative mood states, which in turn reinforces dependence and exacerbates depressive symptoms over time [17]. Conversely, chronic alcohol consumption can precipitate neurochemical changes that heighten vulnerability to depression, suggesting a bidirectional relationship that complicates both diagnosis and treatment [18].

Beyond these immediate mechanisms, it is increasingly recognized that the broader public health context shaped by the COVID-19 pandemic continues to influence the wellbeing of today’s young adults, even several years after its onset. A growing body of research has shown that pandemic-related restrictions, lifestyle disruptions, and prolonged social isolation have left lasting imprints on both mental and physical health. For example, evidence from adolescents in Northern Ireland indicates that lockdowns were associated with worsened mental health, particularly among females and sexual minority youth, with young people reporting heightened psychological distress, concerns about educational outcomes, and a sense of being deprioritized during the crisis [19]. Similarly, studies among Slovak young adults have documented significant pandemic-related declines in physical health markers, including reductions in bone mineral density and bone mineral content [20], alongside notable long-term psychological and physiological symptoms following COVID-19 infection, such as memory difficulties, concentration problems, headaches, decreased physical fitness, and menstrual cycle changes in women [21]. Together, these findings illustrate that the pandemic has created a unique developmental and environmental backdrop for this generation, shaping coping behaviors, stress responses, and overall vulnerability to mental health difficulties, including depressive symptoms and alcohol-related risk behaviors. Incorporating this wider epidemiological context is therefore essential for understanding contemporary student health profiles, as the pandemic represents a generational event with sustained implications for emotional functioning and health-related behaviors.

Despite extensive literature on clinical populations, relatively fewer studies have examined the depression–alcohol nexus within non-clinical university cohorts. Student populations offer a unique context, as they are typically characterized by high-functioning individuals whose risk behaviors may remain subclinical yet still confer long-term mental health consequences [22]. Cross-sectional studies using validated screening instruments such as the Patient Health Questionnaire-9 (PHQ-9) and the Alcohol Use Disorders Identification Test (AUDIT) have revealed striking prevalence rates. Moreover, maladaptive coping strategies, particularly avoidance and emotion-focused coping, have been linked to both increased drinking and heightened depressive symptomatology in student populations [23,24].

Gender differences in the presentation and interplay of these conditions further complicate this picture. Research suggests that male students are more likely to engage in heavy episodic drinking, possibly as a culturally reinforced coping mechanism for stress, while findings regarding internalizing symptoms are mixed, with some studies showing higher rates in females and others finding comparable levels across genders [9]. Some evidence indicates that depressive symptoms in men may manifest more through anhedonia or irritability, whereas women may exhibit more affective and ruminative features [25]. The intersection of gender, alcohol use, and depression thus represents a key area for targeted prevention, as interventions that neglect these distinctions may fail to reach the most at-risk subgroups.

Understanding how depression and hazardous drinking co-occur in university settings carries significant implications for prevention and intervention. Campus-based health programs can play a pivotal role in early identification by employing brief, validated screening tools such as the PHQ-9 and AUDIT to flag at-risk students before clinical thresholds are reached. Furthermore, integrating psychoeducation about alcohol’s impact on mood regulation and cognitive performance into university curricula may mitigate misconceptions about its use as a coping strategy. Evidence suggests that students often underestimate the severity of their drinking behaviors while overestimating the social acceptability of alcohol consumption [7].

Although previous research has explored the association between alcohol use and depression, most studies have focused on clinical or treatment-seeking populations, with far fewer examining non-clinical cohorts of university students using validated psychometric instruments [26,27]. Moreover, the evidence remains inconsistent regarding the moderating role of gender in this relationship, as findings vary across cultural and educational contexts. Importantly, data from Eastern European student populations, where social drinking norms and mental health stigma differ markedly from those in Western settings, are particularly scarce [6].

Therefore, this study aimed to investigate the relationship between alcohol consumption and depressive symptoms among university students, and to explore potential gender differences in this association. By addressing this gap in the literature, the present research contributes region-specific evidence from a Central–Eastern European context to a field largely dominated by Western studies. This regional and cultural perspective is crucial for informing locally adapted, gender-sensitive screening and intervention strategies in university health services.

## 2. Materials and Methods

### 2.1. Study Design and Participants

This study employed a cross-sectional design aimed at exploring the relationship between alcohol consumption and depressive symptoms among university students. Participants were recruited through convenience sampling from the University of Medicine and Pharmacy of Craiova’s Faculty of Medicine between March and June 2024. Recruitment was conducted via email invitations, classroom announcements, and online learning platforms. Interested students were directed to an online survey platform that included an informed consent form, demographic questions, and two standardized psychological assessment instruments: the Alcohol Use Disorders Identification Test (AUDIT) and the Depression, Anxiety, and Stress Scale-21 (DASS-21).

Eligibility criteria required participants to be currently enrolled as full-time or part-time university students and to be at least 18 years of age. Individuals with diagnosed psychiatric conditions other than depression, or those under medical advice restricting alcohol consumption, were excluded to minimize confounding effects. A total of 110 students consented to participate, of whom 7 were excluded for the following reasons: 4 due to medical restrictions on alcohol use, 2 due to pre-existing psychiatric disorders, and 1 due to alcohol allergy. The final analytic sample thus comprised 103 participants. Sample size was determined based on a priori power analysis conducted using G*Power 3.1 (Heinrich-Heine-Universität Düsseldorf, Düsseldorf, Germany). To detect a medium effect size (*r* = 0.30) for a bivariate correlation between alcohol use and depressive symptoms, with α = 0.05 (two-tailed) and statistical power of 0.80, the minimum required sample size was estimated at 84 participants. The final sample of 103 participants exceeded this threshold, ensuring adequate statistical power for the primary analyses.

The Faculty of Medicine at the University of Medicine and Pharmacy of Craiova has approximately 1200 students enrolled in the second year of study during the 2023–2024 academic year. Our sample of 103 participants represents approximately 8.6% of this population. While convenience sampling limits generalizability, the gender distribution in our sample (56.3% female, 43.7% male) is consistent with the overall gender composition of medical faculties in Romania, where female students typically represent 55–60% of enrollment.

Gender identity was self-reported as ‘female,’ ‘male,’ or ‘other.’ No participants selected ‘other,’ and all respondents’ self-identified gender corresponded to their biological sex. Therefore, analyses were conducted using the two self-identified gender categories (female, male), and the term ‘gender’ in this manuscript refers to participants’ self-identified gender.

### 2.2. Measures

#### 2.2.1. Alcohol Use

Alcohol consumption patterns were assessed using the Alcohol Use Disorders Identification Test (AUDIT) developed by the World Health Organization. This ten-item questionnaire identifies hazardous and harmful drinking through three conceptual domains: consumption (items 1–3), dependence symptoms (items 4–6), and adverse consequences or harm (items 7–10). Each item is scored from 0 (never) to 4 (daily or almost daily), yielding a total score ranging from 0 to 40. Based on WHO guidelines, an AUDIT score ≥ 8 indicates hazardous or harmful alcohol use, while scores of 15–19 suggest likely alcohol dependence, and those ≥20 indicate probable severe dependence. The AUDIT has been validated across diverse cultural contexts and demonstrates high internal consistency (Cronbach’s α typically > 0.80) and test–retest reliability [23].

#### 2.2.2. Depression and Psychological Distress

Depressive symptoms were evaluated using the Depression, Anxiety and Stress Scale-21 (DASS-21). The DASS-21 is a brief, 21-item self-report instrument that measures negative emotional states experienced over the past week. Responses are rated on a four-point Likert scale ranging from 0 (did not apply to me at all) to 3 (applied to me very much or most of the time). Scores are summed and multiplied by two to maintain equivalence with the full 42-item version, producing three subscale scores for depression, anxiety, and stress, each ranging from 0 to 42. The depression subscale was the primary variable of interest in this study. Severity levels were categorized as follows: normal (0–9), mild (10–13), moderate (14–20), severe (21–27), and extremely severe (28+). The DASS-21 has demonstrated robust psychometric validity in university populations, with Cronbach’s α values exceeding 0.90 for the depression subscale [28].

#### 2.2.3. Data Collection Procedure

All data were collected anonymously through a secure online platform (Qualtrics^®^, Qualtrics LLC, Provo, UT, USA), which ensured data confidentiality and participant anonymity. Participants provided informed consent electronically prior to beginning the survey. Completion time averaged 12 min. The survey emphasized confidentiality and voluntary participation, with participants free to withdraw at any point without penalty. No personally identifiable information was recorded, ensuring participant anonymity.

#### 2.2.4. Statistical Analysis

Data were analyzed using IBM SPSS Statistics version 29.0 (IBM Corp., Armonk, NY, USA). Descriptive statistics were computed to summarize demographic characteristics and scale distributions. Normality of continuous variables (AUDIT and DASS-21 scores) was assessed via the Shapiro–Wilk test and visual inspection of Q–Q plots. Given that both measures exhibited acceptable skewness (<|1.0|), parametric tests were applied.

Pearson’s correlation coefficient (*r*) was used to examine bivariate associations between alcohol use (AUDIT total score) and depressive symptoms (DASS-21 depression subscale). Independent-samples *t*-tests were conducted to evaluate gender differences in AUDIT and DASS-21 scores. Where data were not normally distributed across subgroups, non-parametric alternatives (Mann–Whitney U tests) were employed. To further assess the predictive relationship between depressive symptoms and hazardous drinking, a binary logistic regression model was performed, with hazardous alcohol use (AUDIT ≥ 8) as the dependent variable and depression severity, gender, and stress level as independent predictors.

Statistical significance was set at *p* < 0.05. Effect sizes (Cohen’s *d* and odds ratios) were reported to facilitate interpretation of results.

#### 2.2.5. Ethical Considerations

The study protocol received ethical approval from the Ethics Committee of the Neuropsychiatry Clinical Hospital of Craiova (Reference No. MED-2024-06, approved on 29 August 2024). All participants provided informed consent electronically before taking part. Data were stored securely and accessed only by the research team. Participants who exhibited high levels of distress or disclosed harmful drinking behaviors were provided with a list of local counseling and support services, including the university’s mental health helpline and student wellbeing office.

During the preparation of this manuscript, the authors used ChatGPT (ScholarAI, GPT-5 model, OpenAI, San Francisco, CA, USA, October 2025) for the purposes of grammar correction, language polishing, graphical concept suggestions, and structuring of scientific content. The authors have thoroughly reviewed and edited all AI-generated outputs and take full responsibility for the final content of this publication. No AI tool was used for generating original research data or conducting formal analyses. 

## 3. Results

### 3.1. Descriptive Statistics

#### 3.1.1. Sample Characteristics

All participants were Romanian second-year university students, aged between 19 and 21 years. The sample comprised 58 females (56.3%) and 45 males (43.7%) (Table 1).

#### 3.1.2. AUDIT Results

AUDIT total scores ranged from 0 to 26 (M = 5.43, SD = 5.80, Median = 3). The distribution was positively skewed (skewness = +1.46), indicating that most participants reported low-to-moderate alcohol consumption, with fewer participants scoring in the higher ranges.

Based on WHO-recommended cut-off criteria, 76.7% (*n* = 79) of participants were classified as low risk (AUDIT 0–7), 14.6% (*n* = 15) as hazardous drinkers (AUDIT 8–15), 4.9% (*n* = 5) as harmful drinkers (AUDIT 16–19), and 3.9% (*n* = 4) as exhibiting possible alcohol dependence (AUDIT ≥ 20). Overall, 23.3% (*n* = 24) of students exceeded the threshold for hazardous alcohol use.

The AUDIT demonstrated excellent internal consistency in this sample (Cronbach’s α = 0.911), exceeding the conventional threshold for high reliability (α > 0.80).

#### 3.1.3. DASS-21 Depression Results

DASS-21 Depression subscale scores ranged from 0 to 40 (M = 13.8, SD = 9.5, Median = 14). The distribution was positively skewed (skewness = +0.84), with nearly half of the sample scoring above the clinical threshold of 14.

When categorized according to standard DASS-21 cut-off criteria, 34.0% (*n* = 35) of students fell within the normal range (0–9), 11.7% (*n* = 12) exhibited mild symptoms (10–13), 30.1% (*n* = 31) moderate symptoms (14–20), 16.5% (*n* = 17) severe symptoms (21–27), and 7.8% (*n* = 8) extremely severe symptoms (≥28). In total, 66.0% (*n* = 68) of participants reported at least mild depressive symptoms.

The DASS-21 Depression subscale demonstrated excellent internal consistency (Cronbach’s α = 0.892). The full 21-item DASS also showed high reliability (α = 0.955) (Table 2).

#### 3.1.4. Gender Differences in AUDIT Scores

Male students reported higher alcohol use severity than female students. Mean AUDIT scores were 6.5 ± 6.1 for males and 3.6 ± 4.2 for females.

An independent-samples *t*-test confirmed that this difference was statistically significant (t(101) = 2.89, *p* = 0.005, mean difference = 2.90, 95% CI [0.88, 4.92]). The effect size was medium (Cohen’s *d* = 0.57, 95% CI [0.17, 0.96]).

When examining risk categories, a higher proportion of male students (33.3%, *n* = 15) exceeded the hazardous drinking threshold (AUDIT ≥ 8) compared to female students (15.5%, *n* = 9).

#### 3.1.5. Gender Differences in DASS-21 Scores

**Figure 1:**
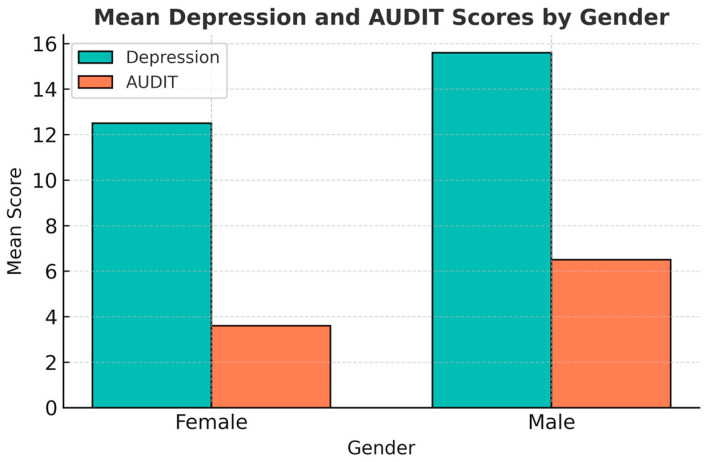
Mean DASS-21 Depression and AUDIT scores by gender. Male students reported higher average levels of both depressive symptoms (M = 15.6) and alcohol use severity (M = 6.5) compared to female students (M = 12.5 and 3.6, respectively).

Male students reported slightly higher depression scores than female students (M = 15.6 ± 9.9 vs. M = 12.5 ± 8.9). However, this difference did not reach statistical significance (t(101) = 1.66, *p* = 0.110, mean difference = 3.10, 95% CI [−0.58, 6.78], Cohen’s *d* = 0.33, 95% CI [−0.06, 0.72]) (Figure 1 and Table 3).

Despite comparable overall depression scores, a significant gender difference emerged for anhedonia. On the DASS-21 item ‘I couldn’t seem to experience any positive feeling at all,’ males were more likely to endorse higher severity levels. Among females, 43.1% reported ‘did not apply at all’ (score = 0) compared to only 26.7% of males. Conversely, 37.8% of males endorsed scores of 2–3 (considerable to most of the time) compared to 12.0% of females. This difference was statistically significant (χ^2^(3) = 8.92, *p* = 0.030) (Table 4).

In summary, while male students exhibited significantly higher alcohol use severity, depressive symptomatology was comparable across genders, with the exception of anhedonia, which was more pronounced in males.

### 3.2. Relationship Between Alcohol Use and Depressive Symptoms

#### 3.2.1. Correlation Analysis

A Pearson correlation confirmed a strong, statistically significant positive relationship between alcohol-use severity and depressive symptoms (*r* = 0.723, 95% CI [0.62, 0.80], *p* < 1 × 10^−17^). Students with higher alcohol use severity consistently exhibited higher levels of depressive symptomatology.

When stratified by gender, the same pattern was observed across both subgroups (Table 5). Among male students (*n* = 45), the correlation between AUDIT and DASS-21 depression scores remained strong (*r* = 0.735, 95% CI [0.56, 0.85], *p* = 1.1 × 10^−6^), with a regression slope of β = 1.20, explaining approximately 43% of the variance in depressive symptoms (*R*^2^ = 0.43). Among female students (*n* = 58), the correlation was also significant (*r* = 0.686, 95% CI [0.52, 0.80], *p* = 2.6 × 10^−6^), with a nearly identical slope (β = 1.22, *R*^2^ = 0.33). Thus, the association between alcohol use and depression was robust across genders, though slightly stronger in males.

#### 3.2.2. Simple Linear Regression

A simple linear regression was performed to examine whether alcohol-use severity predicted depression levels. The model was statistically significant, F(1,101) = 99.47, *p* < 0.001, accounting for approximately 49.6% of the variance in depressive symptoms (*R*^2^ = 0.496).

The estimated regression equation was:

Depression Score=7.23+1.31×AUDIT Score

Each one-point increase in AUDIT total score corresponded to an average 1.31-point increase in depression score (95% CI [1.05, 1.57], *p* < 0.001).

**Figure 2:**
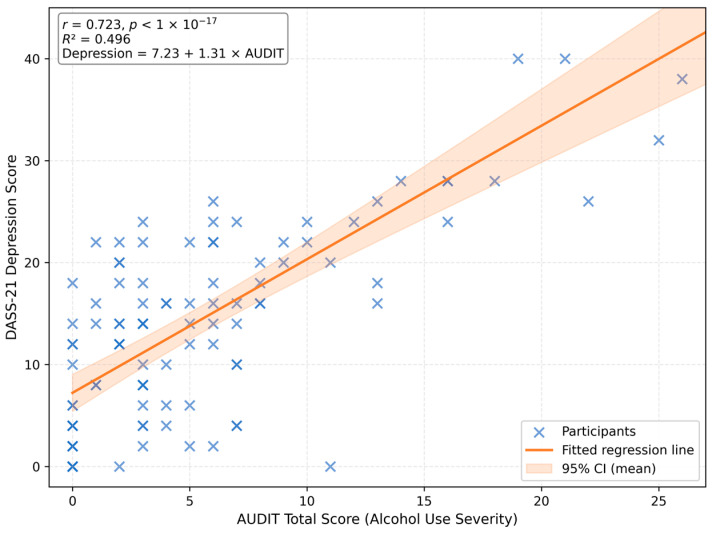
Scatterplot illustrating the relationship between total AUDIT scores and DASS-21 Depression scores among university students (*n* = 103). The solid line represents the fitted simple linear regression (*R*^2^ = 0.496), and the shaded band indicates the 95% confidence interval for the mean prediction. The model equation was Depression = 7.23 + 1.31 × AUDIT (*r* = 0.723, *p* < 1 × 10^−17^).

This model suggests a moderate-to-strong linear association between alcohol use severity and depressive symptomatology (Figure 2).

#### 3.2.3. Multiple Regression Controlling for Gender

To determine whether gender influenced the alcohol–depression relationship, a multiple linear regression was conducted with DASS-21 Depression as the dependent variable and AUDIT total score and gender (0 = female, 1 = male) as predictors. The model was statistically significant (F(2,100) = 55.28, *p* < 0.001), explaining 52.5% of the variance (*R*^2^ = 0.525, Adjusted *R*^2^ = 0.516).

AUDIT remained a significant predictor of depression (B = 1.21, 95% CI [0.97, 1.44], *p* < 0.001), while gender was not significant (B = −0.98, 95% CI [−3.70, 1.74], *p* = 0.477). This finding indicates that the positive association between alcohol use and depression is robust across genders, and that gender does not moderate this relationship (Table 6).

#### 3.2.4. Binary Logistic Regression Predicting Hazardous Alcohol Use

A binary logistic regression was performed to identify predictors of hazardous drinking (AUDIT ≥ 8), with depression severity (DASS-21 Depression score), gender (0 = female, 1 = male), and stress level (DASS-21 Stress score) as independent variables.

The overall model was statistically significant (χ^2^(3) = 43.43, *p* < 0.001), explaining approximately 38.8% of the variance in hazardous drinking (McFadden’s pseudo *R*^2^ = 0.388; Nagelkerke *R*^2^ = 0.519). The model correctly classified 85.4% of cases (sensitivity = 54.2%, specificity = 94.9%).

**Figure 3:**
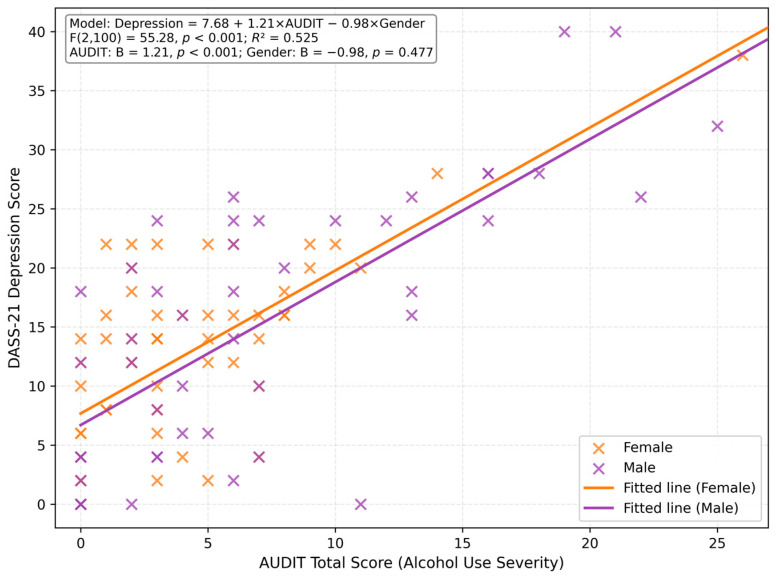
Scatterplot of DASS-21 Depression scores by AUDIT total score, stratified by gender (*n* = 103). Lines show the fitted multiple linear regression controlling for gender (parallel slopes). Model: Depression = 7.68 + 1.21 × AUDIT − 0.98 × Gender (0 = female, 1 = male); F(2,100) = 55.28, *p* < 0.001; *R*^2^ = 0.525. AUDIT was a significant predictor (B = 1.21, *p* < 0.001), while gender was not (B = −0.98, *p* = 0.477).

Depression severity emerged as the only significant predictor of hazardous drinking (B = 0.170, Wald z = 2.43, *p* = 0.015). Each one-point increase in DASS-21 Depression score was associated with a 19% increase in the odds of hazardous drinking (OR = 1.19, 95% CI [1.03, 1.36]). Neither gender (OR = 1.39, 95% CI [0.40, 4.83], *p* = 0.600) nor stress level (OR = 1.08, 95% CI [0.94, 1.23], *p* = 0.298) significantly predicted hazardous drinking when controlling for depression severity (Table 7 and Figure 3).

### 3.3. Supplementary Analyses

#### 3.3.1. Drinking Frequency and Depression

A supplementary analysis explored the relationship between drinking frequency (AUDIT Item 1; coded 0 = “Never” to 4 = “Four or more times a week”) and DASS-21 Depression scores. The results revealed a moderate positive correlation (*r* = 0.552, 95% CI [0.40, 0.67], *p* = 1.5 × 10^−9^), demonstrating that students who reported drinking more frequently also reported higher levels of depressive symptoms.

**Figure 4:**
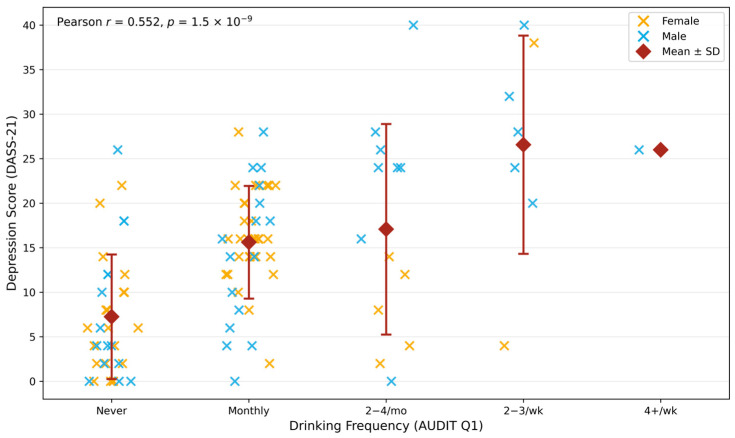
Relationship between depression and drinking frequency among university students (*n* = 103). Each point represents one participant; horizontal jitter indicates overlapping cases. Yellow crosses represent female participants; blue crosses represent male participants. Red (circles) diamonds and error bars represent group means ± SDs. Pearson correlation between drinking frequency (coded ordinally) and DASS-21 Depression score: *r* = 0.552, *p* = 1.5 × 10^−9^. Depression scores increased steadily from “Never” to higher drinking frequency groups.

Overall, higher AUDIT scores were associated with proportionately higher depression scores, with comparable regression coefficients in both sexes (≈1.2 points of depression per 1-point increase in AUDIT). Although the cross-sectional design prevents causal inference, these results suggest that drinking frequency alone is meaningfully linked with depressive symptom severity in this university student population (Figure 4).

#### 3.3.2. Binge Drinking Patterns and Depression

Among the 103 students surveyed, 68 participants (66.0%) reported consuming alcohol at least once per month, indicating that occasional to moderate drinking was common in this sample.

**Figure 5:**
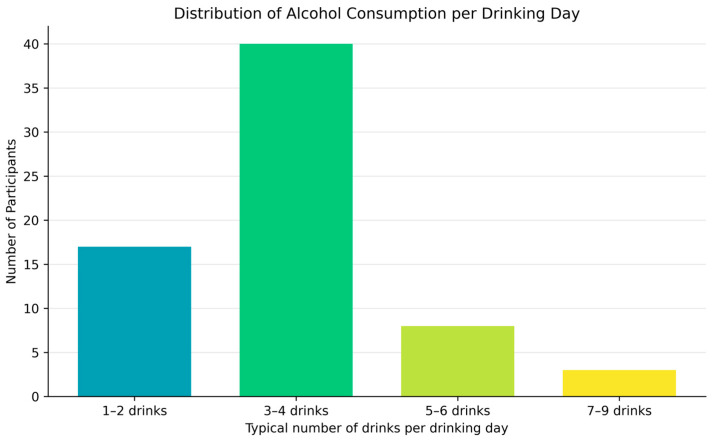
Distribution of the typical number of drinks consumed per drinking occasion among participants who reported alcohol use (*n* = 68). Most students reported consuming 3–4 drinks per occasion, indicating moderate drinking patterns, while a minority engaged in heavier drinking (5 or more drinks).

The distribution of the number of standard drinks typically consumed per occasion is illustrated in Figure 5. The modal category was 3–4 drinks (*n* = 40; 58.8%), followed by 1–2 drinks (*n* = 17; 25.0%), 5–6 drinks (*n* = 8; 11.8%), and 7–9 drinks (*n* = 3; 4.4%).

No participants reported consuming 10 or more standard drinks during a typical drinking episode. This pattern suggests that, while most students engaged in moderate drinking behaviors, a notable minority exhibited episodic heavy drinking patterns.

When asked “How often do you have six or more drinks on one occasion?”, responses indicated that 65.0% (*n* = 67) of students reported *never* engaging in binge drinking, 21.4% (*n* = 22) did so *less than monthly*, 8.7% (*n* = 9) *monthly*, and 4.9% (*n* = 5) *weekly*.

None reported binge drinking *daily or almost daily*. Overall, 13.6% of participants endorsed binge-drinking episodes occurring at least once per month, and 4.9% reported doing so weekly or more often.

#### 3.3.3. Gender Differences in Binge Drinking

Gender-stratified analyses revealed marked differences in binge-drinking frequency.

Among female students (*n* = 58), only 3 participants (5.2%) reported binge drinking at least monthly, compared to 11 of 45 male students (24.4%).

A chi-square test confirmed that this gender difference was statistically significant (χ^2^(2) = 11.19, *p* = 0.004), indicating that monthly or more frequent binge drinking was substantially more prevalent among male students.

This gender disparity aligns with prior research documenting higher rates of risky drinking behaviors among young men in academic populations (Table 8).

The data indicate that most students drink moderately, yet a significant minority engage in binge drinking, a pattern associated with higher psychological distress and potential harm.

The gender gap observed in binge frequency highlights potential differences in drinking norms, coping strategies, and risk perception between male and female students.

These findings underscore the need for targeted harm-reduction and mental health interventions, particularly among male students exhibiting high-risk drinking behaviors.

Mean DASS-21 Depression scores increased progressively with binge-drinking frequency, from 3.6 ± 2.4 among non-binge drinkers to 10.0 ± 6.0 among those reporting weekly binge episodes. Although the small subgroup sizes precluded formal statistical testing, the pattern suggests that heavier episodic drinking is associated with higher depressive symptom levels (Table 9).

## 4. Discussion

Our findings demonstrate a robust positive association between alcohol consumption and depressive symptoms among Romanian university students, with alcohol-use severity explaining nearly half of the variance in depressive symptoms. This suggests that even moderate hazardous drinking may signal clinically relevant emotional distress. This aligns with a growing body of literature on the bidirectional relationship between substance use and mood disorders in young adults [29].

From a mechanistic standpoint, this relationship is supported by both psychological and biological factors. Behaviorally, alcohol is often used as a maladaptive coping strategy to regulate negative affect, academic stress, or social pressures [30]. Neurobiologically, prolonged alcohol misuse disrupts serotonergic and dopaminergic neurotransmission, impairs the HPA axis, and alters neuroplasticity—factors also implicated in depressive pathophysiology [17]. Furthermore, alcohol-related sleep disruption, cognitive decline, and social withdrawal can perpetuate low mood and functional impairment [31,32].

Recent evidence also underscores the enduring impact of the COVID-19 pandemic on student mental health [33]. As noted earlier, pandemic-related circadian and hormonal disruptions may contribute to the observed association [14]. These physiological disruptions may partially explain the co-occurrence of depressive symptoms and alcohol misuse in this population, particularly in post-pandemic student cohorts.

Sociocultural factors likely amplify this relationship in the Romanian context [11]. Students in the region may face additional barriers including mental health stigma and limited support services [34]. These regional particularities underline the importance of context-specific interventions and reinforce the novelty of our findings, as very few studies to date have explored these patterns in post-pandemic, Eastern European student populations.

The strong positive correlation we observed between alcohol consumption and depressive symptoms (*r* = 0.72) is consistent with findings from university student populations worldwide, although our effect size appears larger than those typically reported. Studies among first-year college students in the United States have documented significant associations between depressive symptoms and alcohol consumption, with heavy drinkers exhibiting markedly higher Beck Depression Inventory scores than their peers [35]. Similarly, research among Australian university students demonstrated that hazardous drinking was significantly associated with both depression and anxiety, with female students showing particular vulnerability to mental health problems associated with hazardous alcohol use [36]. A meta-analysis examining 42 cohort studies (*n* = 338,426) confirmed that alcohol use disorders are associated with a 57% increased risk of subsequent depressive symptoms (RR = 1.57, 95% CI 1.41–1.76) [37]. In European contexts, Slovak university students during the COVID-19 pandemic displayed significant positive correlations between perceived stress, depression, and alcohol use disorders, with gender-specific patterns comparable to our findings [38].

With respect to gender, male students in our sample reported significantly higher alcohol-use severity and marginally elevated depressive symptoms, though the latter did not reach statistical significance. Notably, males showed significantly greater anhedonia, which aligns with research suggesting that men may express depressive symptoms through loss of positive affect rather than sadness or guilt. Despite these mean-level differences, the strength of the alcohol–depression association was comparable across genders, indicating that hazardous drinking carries similar emotional correlates regardless of sex [39,40,41]. Research among US college students found that the association between major depressive disorder and higher alcohol intoxication during heavy drinking episodes was actually stronger in female than male students, suggesting that depressed women may be at particular risk when they engage in heavy drinking [9]. Furthermore, studies have indicated that men demonstrate a stronger relationship between psychological distress and weekly alcohol consumption, and that elevated sadness predicts subsequent alcohol use more strongly in male than female college students [9,42]. Conversely, longitudinal research found that depression predicted alcohol problems in women but not in men, with women more likely to experience depression before developing alcohol use disorders whereas men typically develop alcohol problems first [43]. These inconsistencies suggest that cultural context may play a significant role in shaping gender-specific pathways. In our Romanian sample, the comparable strength of the alcohol–depression correlation across genders (males: *r* = 0.74; females: *r* = 0.69) may reflect either shared sociocultural influences on drinking behaviors or the specific characteristics of post-pandemic student populations in Eastern Europe, where traditional gender roles regarding alcohol use may differ from Western contexts.

Emerging research also suggests that sex-specific hormonal fluctuations (e.g., testosterone, estrogen) may modulate reward sensitivity and drinking behavior, yet our study did not assess biological factors. Future research integrating hormonal, neuroendocrine, and chronobiological variables may help elucidate the biological underpinnings of gendered coping mechanisms.

This study’s strengths include its use of psychometrically validated tools (AUDIT, DASS-21), its high internal consistency (α = 0.91; α = 0.89), and its focus on non-clinical young adults, a population often overlooked in mental health surveillance. However, the cross-sectional design limits causal inference, and self-report measures may be prone to bias. Additionally, confounders such as trauma history, academic workload, or concurrent anxiety symptoms were not assessed and may partly account for the observed relationships. Perceived and received social support were not directly assessed either, which limits conclusions regarding the potential buffering role of social support in the association between stress, depressive symptoms, and alcohol use.

Moving forward, longitudinal studies should explore causal pathways between mood symptoms and alcohol use, while also incorporating biomarkers (e.g., cortisol levels, sleep actigraphy) and psychosocial mediators (e.g., peer norms, coping styles). Preventive interventions that target both emotional wellbeing and alcohol use, ideally adapted to regional cultural norms and post-pandemic realities, may prove especially effective in supporting vulnerable student populations.

### Limitations

Although coping strategies were not directly measured, alcohol use, particularly hazardous and binge drinking, may be interpreted as a maladaptive coping behavior within the self-medication framework. However, the absence of validated coping-style instruments (e.g., Brief COPE) limits the ability to distinguish between adaptive and maladaptive stress-response strategies. Similarly, risk awareness and health literacy were not directly assessed, which constrains conclusions regarding participants’ understanding of alcohol-related and mental-health risks.

Alcohol consumption was assessed via self-report and may therefore be subject to recall bias or social-desirability effects, despite the use of a well-validated screening instrument. In addition, cultural norms surrounding alcohol use and mental-health stigma in Central–Eastern Europe may have influenced both drinking behaviors and symptom reporting, potentially limiting the generalizability of the findings to other cultural contexts. Finally, several potential confounding factors, including socioeconomic status, sleep quality, anxiety symptoms, lifestyle behaviors, and prior mental health history, were not fully assessed and may have contributed to the observed associations.

## 5. Conclusions

This study found a strong positive correlation (*r* = 0.72) between alcohol consumption and depressive symptoms among Romanian university students, with alcohol use severity explaining nearly half (49.6%) of the variance in depression scores. Two-thirds of students (66%) reported at least mild depressive symptoms, and nearly one-quarter (23.3%) exceeded the threshold for hazardous alcohol use. Male students reported significantly higher alcohol consumption than females (*p* = 0.005), yet the alcohol–depression association was similarly strong across both genders. Depression severity emerged as the only significant predictor of hazardous drinking in logistic regression analyses, with each one-point increase in DASS-21 Depression score associated with a 19% increase in the odds of hazardous alcohol use. Notably, male students exhibited significantly greater anhedonia than females, suggesting potential gender differences in depressive symptom presentation. These findings confirm the co-occurrence of hazardous drinking and depressive symptoms in a non-clinical, post-pandemic Eastern European student population.

### Recommendations

Based on these findings, we recommend the implementation of routine, integrated screening for both depressive symptoms and alcohol use in university health services, using validated instruments such as the AUDIT and DASS-21. Given the strong predictive relationship between depression severity and hazardous drinking observed in this study, early identification of depressive symptoms may help identify students at risk for problematic alcohol use. The gender differences in alcohol consumption and anhedonia suggest that gender-sensitive screening and intervention approaches may be warranted. University wellbeing programs should consider dual-focus strategies addressing both emotional distress and alcohol use simultaneously to support at-risk student populations.

## Figures and Tables

**Table 1:** Demographic characteristics of the study participants.

Variable	Category/Description	*n* (%) or Range
Gender	Female	58 (56.3%)
	Male	45 (43.7%)
Age	Range	19–21 years
Nationality	Romanian	100%
Year of study	Second year (undergraduate level)	100%

**Table 2:** Descriptive statistics of AUDIT and DASS-21 Depression scores (*n* = 103).

Variable	Range	Mean ± SD	Median	Skewness	Interpretation
AUDIT Total	0–26	5.43 ± 5.80	3	+1.46	Positively skewed, most ≤ 8
DASS-21 Depression	0–40	13.8 ± 9.5	14	+0.84	Right-skewed, half ≥ 14

AUDIT = Alcohol Use Disorders Identification Test; DASS-21 = Depression, Anxiety, and Stress Scales–21. Values represent descriptive statistics for the full sample (*n* = 103). Skewness values > 0 indicate right-skewed distributions, reflecting that most participants reported low-to-moderate alcohol consumption and depressive symptom scores, with fewer participants scoring in the higher ranges.

**Table 3:** Gender Differences in AUDIT and DASS-21 Depression Scores.

Variable	Gender	*n*	Mean ± SD	Mean Difference	95% CI for Difference	t(df)	*p*-Value	Cohen’s *d*	95% CI for *d*	Interpretation
AUDIT Total	Male	45	6.5 ± 6.1	2.90	[0.88, 4.92]	2.89 (101)	0.005	0.57	[0.17, 0.96]	Significantly higher in men
	Female	58	3.6 ± 4.2	-	-	-	-	-	-	-
DASS-21 Depression	Male	45	15.6 ± 9.9	3.10	[−0.58, 6.78]	1.66 (101)	0.110	0.33	[−0.06, 0.72]	ns (no significant difference)
	Female	58	12.5 ± 8.9	-	-	-	-	-	-	-

Note: *n* = number of participants; SD = standard deviation; df = degrees of freedom; CI = confidence interval; ns = non-significant. AUDIT = Alcohol Use Disorders Identification Test; DASS-21 = Depression, Anxiety, and Stress Scales-21. Mean difference = Males − Females. Cohen’s d effect size interpretation: small (0.2–0.5), medium (0.5–0.8), large (>0.8).

**Table 4:** Distribution of responses to the DASS-21 item *“I couldn’t seem to experience any positive feeling at all”* by gender.

Response Category (“I Couldn’t Seem to Experience Any Positive Feeling at All”)	Female (*n* = 58)	Male (*n* = 45)
0—Did not apply at all	25 (43.1%)	12 (26.7%)
1—Applied to some degree	26 (44.8%)	16 (35.6%)
2—Applied to a considerable degree	5 (8.6%)	14 (31.1%)
3—Applied most of the time	2 (3.4%)	3 (6.7%)

Note. Percentages reflect the proportion of participants within each gender group selecting each response option. Male students were more likely than female students to endorse higher-severity responses (categories 2–3), indicating greater anhedonic symptomatology.

**Table 5:** Relationship between Alcohol Use Severity and Depressive Symptoms by Gender.

Group	*n*	Pearson’s *r*	95% CI for *r*	*p*-Value	β (Slope)	95% CI for β	*R* ^2^	Interpretation
All participants	103	0.723	[0.62, 0.80]	<1 × 10^−17^	1.31	[1.05, 1.57]	0.52	Strong positive association
Males	45	0.735	[0.56, 0.85]	1.1 × 10^−6^	1.20	[0.82, 1.58] *	0.43	Strong positive association
Females	58	0.686	[0.52, 0.80]	2.6 × 10^−6^	1.22	[0.80, 1.64] *	0.33	Strong positive association

Note. β = unstandardized regression coefficient (Depression = α + β × AUDIT). Depression was measured using the DASS-21 depression subscale (range = 0–42). CI = confidence interval. * CI for β in gender subgroups estimated from standard errors derived from *R*^2^ and sample size.

**Table 6:** Multiple Linear Regression Predicting DASS-21 Depression Scores from AUDIT and Gender (*n* = 103).

Predictor	B (Unstandardized)	SE	t	*p*	95% CI for B	Interpretation
Constant	7.68	0.98	7.81	<0.001	[5.73, 9.64]	Baseline depression when AUDIT = 0
AUDIT Total	1.21	0.12	10.24	<0.001	[0.97, 1.44]	↑ 1 AUDIT pt → ↑ 1.21 Depression pts
Gender (Male = 1)	−0.98	1.37	−0.71	0.477	[−3.70, 1.74]	ns (no gender effect)

Model statistics: F(2, 100) = 55.28, *p* < 0.001; *R*^2^ = 0.525; Adjusted *R*^2^ = 0.516. Note. Dependent variable = DASS-21 Depression Score. Predictors = AUDIT Total Score; Gender (0 = female, 1 = male). ns = non-significant. CI = confidence interval.

**Table 7:** Binary Logistic Regression Predicting Hazardous Alcohol Use (AUDIT ≥ 8) from Depression, Gender, and Stress (*n* = 103).

Predictor	B	SE	Wald z	*p*	OR	95% CI
Intercept	−5.578	1.15	−4.84	<0.001	0.00	[0.00, 0.04]
Depression severity	0.170	0.07	2.43	0.015	1.19	[1.03, 1.36]
Gender (Male = 1)	0.332	0.63	0.52	0.600	1.39	[0.40, 4.83]
Stress level	0.073	0.07	1.04	0.298	1.08	[0.94, 1.23]

Model: χ^2^(3) = 43.43, *p* < 0.001; McFadden’s *R*^2^ = 0.388; Nagelkerke *R*^2^ = 0.519; Accuracy = 85.4%.

**Table 8:** Frequency and Quantity of Alcohol Consumption Among Monthly Drinkers (*n* = 68).

Drinking Pattern	Category	*n* (%)	Interpretation
Typical number of drinks per occasion	1–2	17 (25.0%)	Light consumption
	3–4	40 (58.8%)	Moderate consumption (most common)
	5–6	8 (11.8%)	Episodic heavy use
	7–9	3 (4.4%)	High-risk pattern
Binge drinking frequency (≥6 drinks/occasion)	Never	67 (65.0%)	No binge drinking
	Less than monthly	22 (21.4%)	Occasional
	Monthly	9 (8.7%)	Regular binge pattern
	Weekly	5 (4.9%)	Persistent high risk

**Table 9:** Mean DASS-21 Depression Scores by Binge Drinking Frequency.

Binge Drinking Frequency (≥Six Drinks per Occasion)	Mean DASS-21 Depression Score	SD	*n*
Never	3.61	2.43	67
Less than monthly	6.64	3.24	22
Monthly	7.22	2.95	9
Weekly	10.00	5.96	5
Daily or almost daily	—	—	0

Note. Mean depression scores were calculated using the DASS-21 Depression subscale. Although small subgroup sizes precluded formal statistical testing, mean values show a positive trend between binge-drinking frequency and depressive symptom severity.

## Data Availability

Dataset available on request from the authors.

## Abbreviations

The following abbreviations are used in this manuscript:

AUDIT	Alcohol Use Disorders Identification Test
B	Unstandardized Regression Coefficient
β	Beta (Regression Coefficient)
CI	Confidence Interval
DASS-21	Depression, Anxiety, and Stress Scale–21
df	Degrees of Freedom
HPA	Hypothalamic–Pituitary–Adrenal (axis)
IBM SPSS	International Business Machines Statistical Package for the Social Sciences
M	Mean
ns	Non-significant
PHQ-9	Patient Health Questionnaire–9
*p*	Probability Value (Statistical Significance)
*r*	Pearson’s Correlation Coefficient
*R*^2^	Coefficient of Determination
SD	Standard Deviation
SE	Standard Error
WHO	World Health Organization
α	Cronbach’s Alpha (Measure of Internal Consistency/Reliability)
